# Serum Biomarkers in Bladder Cancer: NMR Metabolomics for Identification and Monitoring during Platinum-Based Therapy

**DOI:** 10.32604/or.2026.068896

**Published:** 2026-03-23

**Authors:** Roberta Giorgione, Daniela Grasso, Elisabetta Gambale, Federico Scolari, Virginia Rossi, Fabrizio Di Maida, Marinella Micol Mela, Barbara Marzocchi, Laura Doni, Adriano Pasqui, Andrea Minervini, Enrico Caliman, Sergio Serni, Andrea Bernini, Serena Pillozzi, Lorenzo Antonuzzo

**Affiliations:** 1 Oncology Unit, Careggi University Hospital, Florence, Italy; 2 Department of Biotechnology, Chemistry and Pharmacy, University of Siena, Siena, Italy; 3 Department of Biomedical, Experimental and Clinical Sciences, University of Florence, Florence, Italy; 4 Unit of Urology and Andrology, Careggi University Hospital, Florence, Italy; 5 Department of Experimental and Clinical Medicine, University of Florence, Florence, Italy; 6 Unit of Urology and Renal Transplantation, Careggi University Hospital, Florence, Italy

**Keywords:** Metabolomics, biomarkers, bladder cancer (BC), chemotherapy

## Abstract

**Objectives:**

To date, predictive and prognostic biomarkers for Bladder Cancer (BC) remain lacking. Existing literature underscores the potential of metabolomics as a valuable tool for biomarker identification. The primary objective of this study is to characterize the serum metabolic profile of BC patients undergoing platinum-based chemotherapy (Pt-CT) to identify potential biomarkers.

**Methods:**

In this pilot study, we investigated the metabolomic profiles of 14 BC patients undergoing Pt-CT in different settings. We compared their baseline profiles with those of healthy controls and tracked key metabolites throughout chemotherapy cycles. Metabolomics profiling was conducted using nuclear magnetic resonance (NMR) spectroscopy. All experiments were performed on a Bruker Avance™ 600 spectrometer.

**Results:**

Serum samples of BC patients had elevated levels of acetate, acetone, hypoxanthine, trimethylamine N-oxide (TMAO), glutamate, lactate, phenylalanine, and ornithine. Conversely, there were decreased levels of carnitine, choline, betaine, aspartate, threonine, 2-hydroxybutyrate, 2-aminobutyrate and histidine when compared with healthy controls. Throughout the CT course, hypoxanthine, glutamate, and aspartate levels increased, while acetone, acetate and TMAO levels decreased.

**Conclusions:**

The results of our study confirm perturbations in several metabolic pathways in the serum samples of BC patients, including glycolysis, fatty acid, purine, and amino acid metabolism. Additionally, TMAO may contribute to BC development by fostering a pro-inflammatory and oxidative stress state. Furthermore, monitoring these metabolites could serve as a valuable tool for predicting treatment response. To the best of our knowledge, no metabolomic studies have assessed BC patients undergoing CT with longitudinal monitoring to identify changes in the metabolic profile induced by treatment.

## 1 Introduction

Worldwide, bladder cancer (BC) is the tenth most common cancer, associated with substantial morbidity and mortality [1]. Muscle-invasive bladder cancer (MIBC) makes up 25% of BC cases [1]. Despite ongoing efforts to improve outcomes in both localized and advanced stages of the disease the absence of reliable prognostic and predictive biomarkers remains a major challenge. Many attempts have been made to molecularly classify BC, resulting in an international consensus that defines six distinct molecular subtypes: papillary luminal (LumP), luminal unspecified (LumNS), luminal unstable (LumU), stroma-rich, basal/squamous (Ba/Sq), and neuroendocrine-like urothelial carcinoma. Specific gene signatures, microenvironment features, genomic alterations, histological variants, morphological patterns, clinical features, treatment options, and prognosis characterize each subtype [2]. However, despite its potential, this classification has limited clinical use, emphasizing the need for further research to bridge the gap between molecular and clinical traits and to identify predictive biomarkers for treatment response [2].

Metabolomics involves studying small-molecule chemical entities, known as metabolites, in biological samples such as urine or blood. These metabolites play a key role in regulating both catabolic and anabolic pathways. Besides being byproducts of genes and proteins, metabolites actively interact with the genome and proteome, serving as both biomarkers and regulators of biological processes [3]. Notably, they contribute to covalent chemical modifications of DNA and RNA (e.g., methylation) as well as proteins (post-translational modifications), thereby affecting essential cellular functions [3]. As a result, metabolomics enables the identification of tumor-specific metabolic biomarkers with potential diagnostic, prognostic, or predictive value. This approach has been successfully applied to various cancers, including breast [4], ovary [5], kidney [6], prostate [7], colorectal [8], and hepatocellular carcinoma [9].

Metabolomics studies in BC have shown promising results in non-invasive biomarker identification. Different disease models are used to study the BC metabolome, including *in vitro* tumor cell cultures, *ex vivo* neoplastic bladder tissues, and human biofluids (blood, urine) [10]. Each model has its pros and cons: *in vitro* cell cultures are less complex but pose challenges when applying findings to *in vivo* systems. *Ex vivo* tumor tissues provide valuable insights into altered metabolites in solid tumors and their microenvironments. However, obtaining these tissues requires invasive surgical procedures, specialized equipment, and expertise. Additionally, tissue samples are often limited in availability, exhibit significant heterogeneity, and may be contaminated by surrounding cells. In contrast, urine is a particularly advantageous specimen in BC research due to its non-invasive collection, ease of handling, stability, and rich metabolite profile, making it widely used in metabolomic studies. Nonetheless, urine composition can be affected by various factors, including clinical conditions, genetics, race, age, gender, lifestyle, diet, and medications. In contrast, blood serum/plasma is an important sample because it directly reflects metabolic processes within the body and is less affected by diurnal variations and confounding factors. Therefore, its composition is considered more standardized than that of urine [10].

Based on the available data, the metabolic signature of BC is mainly characterized by changes in metabolites linked to energy metabolic pathways, especially glycolysis [11–14], amino acid metabolism [11,12,15], and fatty acid metabolism [16], which are known to be essential for cell proliferation, as well as glutathione metabolism, vital for maintaining cellular redox balance [17]. Additionally, impairments in purine and pyrimidine metabolism [15,18,19] have been identified in this tumor. Some of these metabolites hold promise as potential biomarkers for early bladder cancer diagnosis, illuminating the primary deregulated metabolic pathways in this neoplasm. Moreover, specific metabolite levels can differentiate between early and advanced stages of malignancy [13,14,16]. In the study by Feng et al., the metabolomic profiles of basal and luminal bladder cancer subtypes were analyzed using a classifier based on transcriptome expression. Among the 133 metabolites evaluated, glycerophosphocholine, hydroxy acids, nucleosides, imidazoles, and pyrimidine nucleosides were identified as differential metabolites, which distinguish between basal and luminal subtypes [18].

The main goal of this study was to analyze the metabolome of patients with urothelial carcinoma of the bladder who are undergoing platinum-based chemotherapy (Pt-CT) The specific objectives were: (i) to characterize the serum metabolic profile of patients with urothelial carcinoma compared to healthy individuals, (ii) to identify changes in the metabolomic profile caused by chemotherapy treatment, (iii) to evaluate the associated altered metabolic pathways, and (iv) to identify potential prognostic and predictive biomarkers of response to chemotherapy.

## 2 Materials and Methods

### 2.1 Study Design and Biological Samples

In this prospective, observational, single-center study, we enrolled 14 adult patients at the Oncology Department of the Careggi University Hospital, aged 68 on average (see Table 1) with a histological diagnosis of urothelial carcinoma of the bladder undergoing Pt-CT. The allowed regimens included Cisplatin-Gemcitabine (CG) or Carboplatin-Gemcitabine (CaG) (day 1–8 every 21 days). The indication for treatment followed the recommendations provided by the Guidelines for urothelial carcinoma of the Italian Association of Medical Oncology (AIOM) [20].

**Table 1:** Clinical-pathological characteristics

Characteristics	Total *n*. 14 (%)	Metastatic Disease *n*. 3 (%)	Adjuvant Therapy *n*. 3 (%)	Neoadjuvant Therapy *n*. 8 (%)
Age—mean (range)	68 (59–82)	76 (72–82)	66 (60–70)	65 (59–71)
Male	13 (92.9)	3 (100.0)	3 (100.0)	7 (87.5)
PS sec ECOG 0	14 (100.0)	3 (100.0)	3 (100.0)	8 (100.0)
BMI, median (range)—kg/m^2^	25 (21–34)	23 (22–24)	24 (22–25)	28 (21–34)
HG urothelial carcinoma	13 (92.9)	3 (100.0)	2 (66.7)	8 (100.0)
Squamous carcinoma	1 (7.1)	-	1 (33.3)	–
Other histological subtypes	5 (35.7)	2 (66.7)	1 (33.3)	2 (25.0)
cT sec. AJCC—8th edition				
cT2	4 (28.6)	1 (33.3)	–	3 (37.5)
cT3	8 (57.1)	–	3 (100.0)	5 (62.5)
cT4	2 (14.3)	2 (66.7)	–	–
cN sec. AJCC—8th edition				
cN0	13 (92.9)	2 (66.7)	3 (100.0)	8 (100.0)
cNx	1 (7.1)	1 (33.3)	–	–
Clinical Stage sec. AJCC—8th edition				
II	3 (21.4)	–	–	3 (37.5)
IIIA	8 (57.1)	–	3 (100.0)	5 (62.5)
IV	3 (21.4)	3 (100.0)	–	–
Previous treatments for NMIBC	3 (21.4)	1 (33.3)	–	2 (25.0)
Chemotherapy				
CG	11 (78.6)	–	3 (100.0)	8 (100.0)
CaG	3 (21.4)	3 (100.0)	–	–
N. of cycles				
1	5 (35.7)	1 (33.3)	2 (66.7)	2 (25.0)
2	1 (7.0)	1 (33.3)	–	0 (0)
3	4 (28.6)	–	1 (33.3)	3 (37.5)
4	4 (28.6)	1 (33.3)	–	3 (37.5)

Note: AJCC, American Joint Committee on Cancer; BMI, Body Mass Index; CaG, Carboplatin-Gemcitabine; CG, Cisplatin-Gemcitabine; HG, high Grade; NMIBC, Non-Muscle Invasive Bladder Cancer; PS: Performance Status; ECOG, Eastern Cooperative Oncology Group; –: zero.

A venous blood sample was collected from each patient before the start of chemotherapy (time 0, T0) and preceding the initiation of each subsequent cycle of chemotherapy, approximately every 21 days (T1 at 21 days from baseline, T2 at 42 days from baseline, T3 at 63 days from baseline). All samples from the control group (CNTR) were collected at baseline.

**Figure 1:**
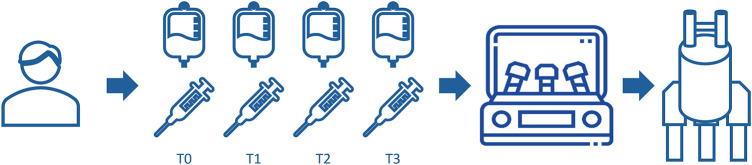
Study design. For each patient, a sample was collected at different time points: T0: time 0 (baseline), before the start of chemotherapy; T1: time 1, at 21 days from baseline; T2: time 2, at 42 days from baseline; T3: time 3, at 63 days from baseline. All samples from the control group (CNTR) were collected at baseline

One 10 mL lavender top Ethylenediaminetetraacetic acid (EDTA) tube (BD Vacutainer^®^ Blood Collection Tubes, BD, Plymouth, UK) and two 5 mL gold top serum separation tubes (BD Vacutainer^®^ Blood Collection Tubes, BD, Plymouth, UK) were collected for each patient. Whole blood was transferred from the EDTA tube into a cryovial using a 10 mL pipette; subsequently, the samples were processed by centrifugation at 2500 rpm at 18°C for 10 min. Subsequently, 2 cryovials of plasma (from EDTA tube) and 2 cryovials of serum (from serum separation tubes) were obtained by transferring the supernatant (Fig. 1). Serum samples for Nuclear Magnetic Resonance (NMR) analysis were aliquoted and immediately stored at −80°C. A control group consisting of 19 healthy individuals, matched in age and sex, was established for NMR profiling. For this purpose, a blood sample was also taken from these individuals. Whole blood was collected in gold top serum separation tubes and processed in the same way as the patients. This resulted in a cryovial of serum for each individual in the control group.

### 2.2 Metabolomic Analysis

#### 2.2.1 NMR Sample Preparation

Dipotassium hydrogen phosphate (K2HPO4), monopotassium phosphate (KH2PO4), and trimethylsilylpropionic acid-d4 (TSP) have been purchased from Merck (Merck KGaA, Darmstadt, Germany). Deionized water was purified using a Milli-Q^®^ System from Millipore (Burlington, MA, USA). A stock solution of phosphate buffer 500 mM (PB) was prepared from K2HPO4 and KH2PO4, and pH was adjusted to 7.0 with sodium hydroxide (NaOH) 1M. NMR samples were prepared with 300 µL of serum, 180 µL of deionised water, 60 µL PB (final concentration of 50 mM), and 60 µL formate 45 mM (final concentration of 4.5 mM) as internal standard. The total deuterium concentration in the samples was maintained at 10% for NMR purposes.

#### 2.2.2 ^1^H NMR Spectroscopy and Data Processing

Metabolomics profiling was obtained by nuclear magnetic resonance (NMR) spectroscopy. All experiments were performed on a Bruker Avance™ 600 spectrometer operating at 14.1 T over a spectral width of 10 kHz. All spectra were obtained with 16 scans, digitised over 32k points, and zero-filled to 128k. Solvent signal removal was achieved with a presaturation power of 55 dB during delay d1 (4 s). A PROJECT (Periodic Refocusing of J Evolution by Coherence Transfer) pulse sequence was used with an echo time (τ) of 0.3 ms with 128 loops to achieve a T2 filter delay of 153.6 ms to obtain an optimal suppression of protein signals [21,22]. NMR data were processed with TopSpin 4.0.8 (Bruker Biospin, Fällanden, Switzerland) software, and metabolites were identified and quantified with Chenomx 9.02 (Chenomx, Edmonton, Canada). Formate (Merck, Darmstadt, Germany) at 4.5 mM concentration was used as an internal standard to calculate metabolite concentrations [21]. We used the Chenomx library to identify and quantify a total of 50 metabolites as µM concentrations (see Fig. S1).

#### 2.2.3 Data Analysis and Visualization

All statistical analyses were carried out with Metaboanalyst 6.0 [21]. The Volcano Plot was used to identify and highlight only the significant metabolites (*p* < 0.05). Multidimensionality reduction analysis on the five datasets of metabolic profiles was conducted using sparse partial least squares discriminant analysis (sPLS-DA). Boxplot figures were generated using GraphPad Prism version 9.5.1 (GraphPad Software, Boston, MA, USA).

### 2.3 Study Oversight

The study protocol was approved by the Careggi University Hospital Ethics Committee (approval number: 22712_bio), and written informed consent was obtained from all study participants prior to participation. All procedures were performed in accordance with the recommendations of the Declaration of Helsinki.

## 3 Results

14 BC patients (aged 68 on average) have been enrolled in the study since July 2022: 3 of them received treatment for metastatic disease, 3 underwent adjuvant chemotherapy, and 8 received neoadjuvant treatments. The clinical-pathological characteristics of patients are summarized in Table 1. Ninety-three percent (92.9%) of the patients were male and had high-grade urothelial carcinoma. All patients had an Eastern Cooperative Oncology Group (ECOG) performance status (PS) of 0 at the time of diagnosis. At diagnosis, 3 patients (21.4%) were at stage II, 8 (57.1%) at stage IIIA, and 3 (21.4%) at stage IV. Three patients had received previous treatments for a non-muscle-invasive bladder cancer (NMIBC). Regarding platinum-based treatment, 3 patients (those with advanced disease) received a combination of carboplatin and gemcitabine, while the remaining 11 received a cisplatin-based combination. At the time of analysis, 5 patients had undergone only 1 cycle of therapy, 1 patient had completed 2 cycles, 4 patients had completed 3 cycles, and 4 patients had completed 4 cycles.

**Figure 2:**
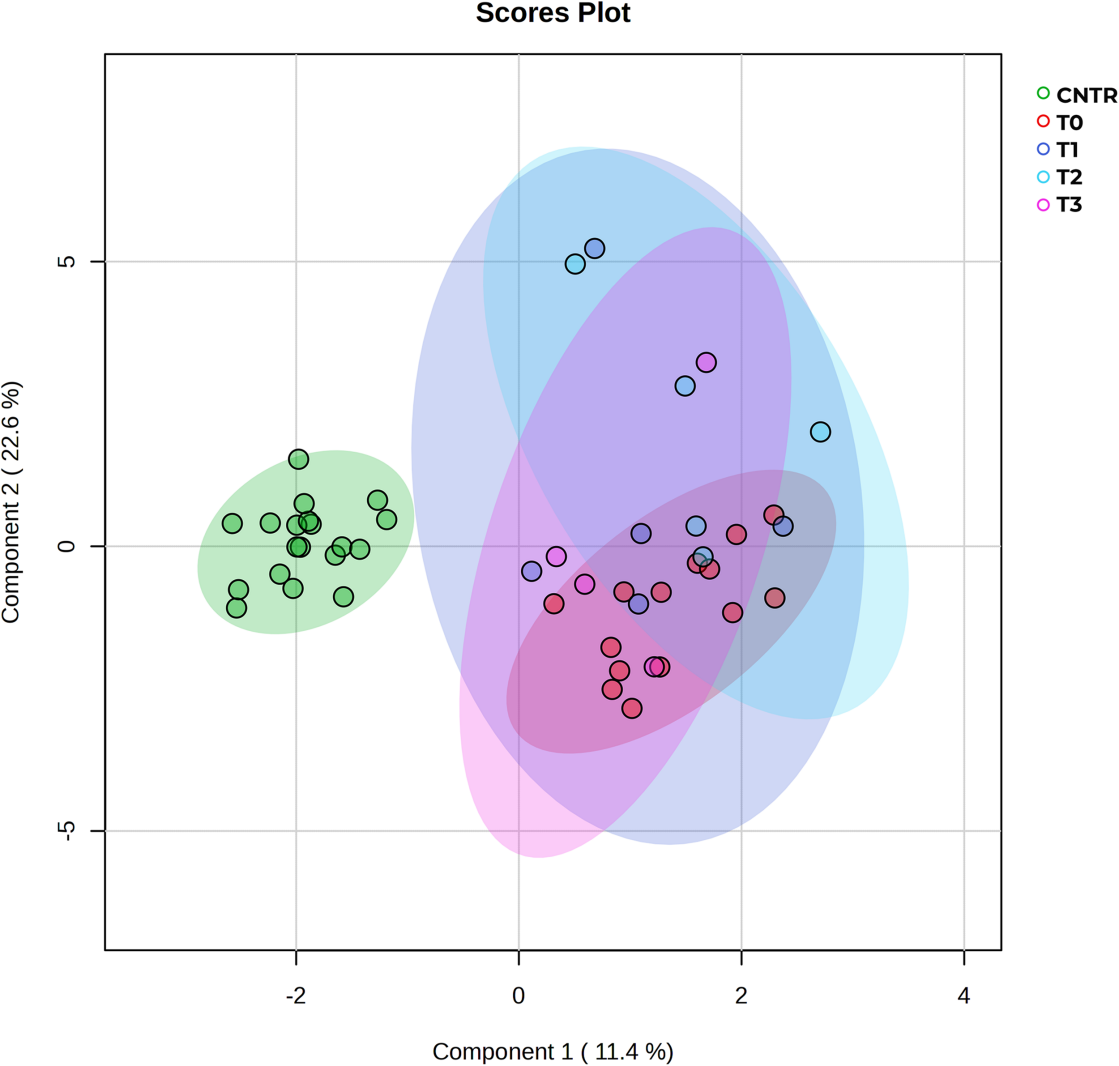
sPLS-DA of controls vs. patients at all times. The score plot shows distinct clustering of control samples (green) vs. bladder cancer patient subgroups (T0–T3). Clear separation of the control group from all tumor groups indicates strong metabolic discrimination, while overlapping clusters among T0–T3 suggest shared metabolic profiles with possible progression-related trends

NMR analysis of patients at T0 was conducted on serum samples collected before chemotherapy began (*n* = 14). The follow-up samples at T1, T2, and T3 (collected at 21, 42, and 63 days from baseline) and those of controls were analysed in the same way. Multidimensionality reduction analysis on the five datasets of metabolic profiles was conducted using sparse partial least squares discriminant analysis (sPLS-DA) and is presented in Fig. 2. The sPLS-DA combines PLS with sparsity (Lasso-style) penalization to perform both dimensionality reduction and variable selection simultaneously, making it well-suited for small datasets when the number of features exceeds the number of samples [22]. The result presents a net metabolic shift in pre-treatment patients (T0) compared to controls. Also, T1 shows limited intra-group variance that increases as treatment progresses (T1–T3), mirroring the variation in individuals’ responses to chemotherapy.

**Figure 3:**
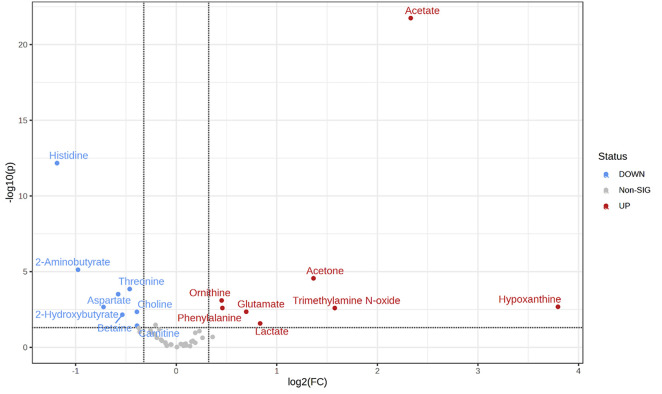
Volcano plot of the fold change vs. *p*-value for the metabolites. Significant metabolites (*p* < 0.05) are labelled and coloured according to the upregulation (red) or downregulation (blue) for BC vs. healthy controls. The binary logarithm of fold change is used to increase the dynamic range

**Figure 4:**
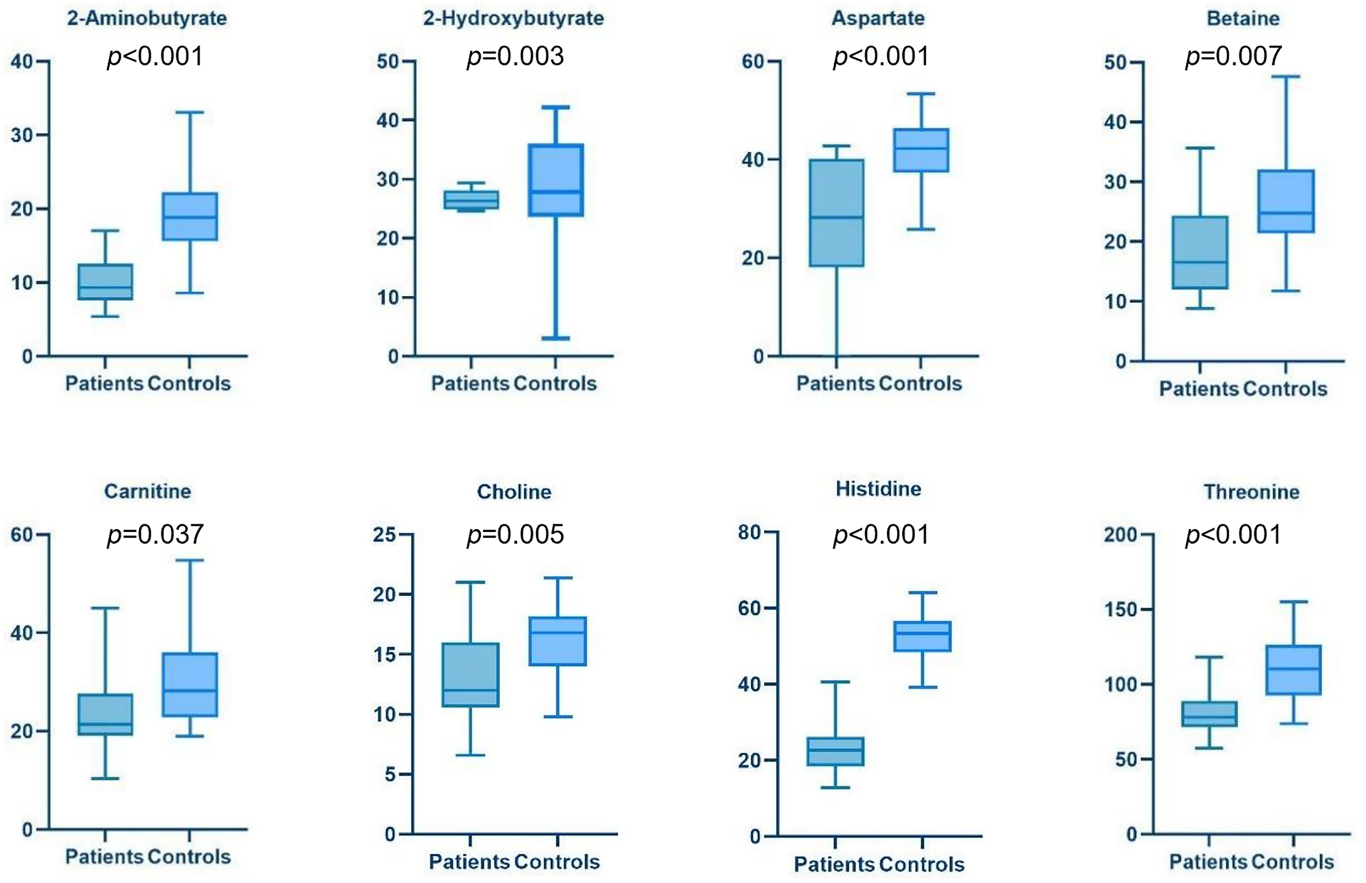

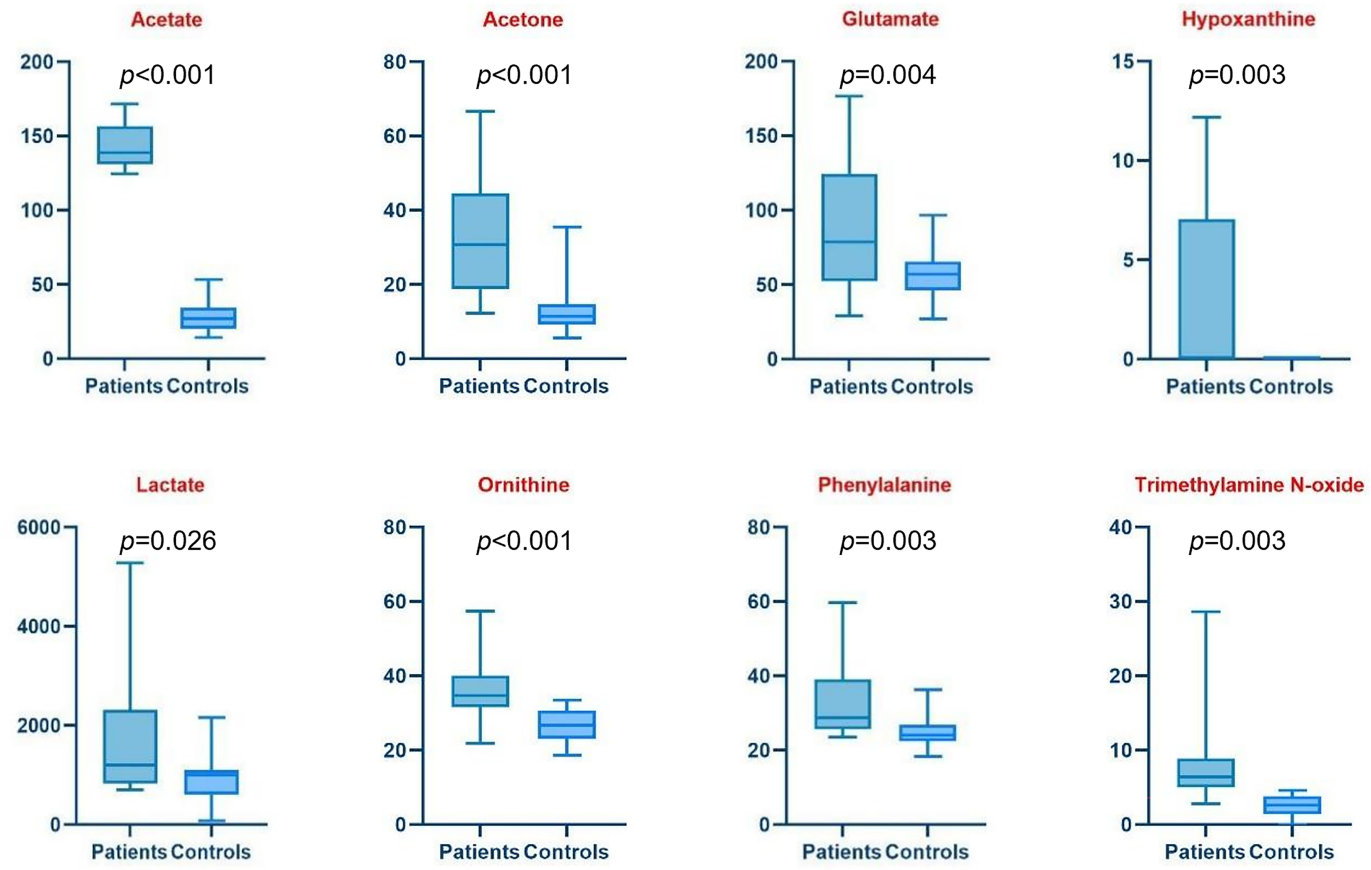
Boxplots of Bladder Cancer patients vs. Controls s show in more detail the trend of each metabolite down- (blue) and up- (red) regulated. The *y*-axis refers to the concentration (µM) of metabolites

The low dispersion observed in the metabolic profiles of controls and T0 (pre-treatment) enables a direct comparison between the two groups. The volcano plot (Fig. 3) highlights metabolites with a fold change (FC) greater than 1.25 (log2FC = 0.33) and a *p*-value < 0.05. Among the upregulated metabolites in BC samples, compared to healthy donor samples, are acetate, acetone, hypoxanthine, trimethylamine N-oxide (TMAO), glutamate, lactate, phenylalanine, and ornithine. Conversely, downregulated metabolites in BC samples include carnitine, choline, betaine, aspartate, threonine, 2-hydroxybutyrate, 2-aminobutyrate, and histidine. A detailed comparison of these dysregulated metabolites is reported as box plots in Fig. 4.

However, the same profiles showed no significant difference when comparing treatment settings (neoadjuvant, adjuvant, metastatic) among patients with BC (see Fig. S2).

**Figure 5:**
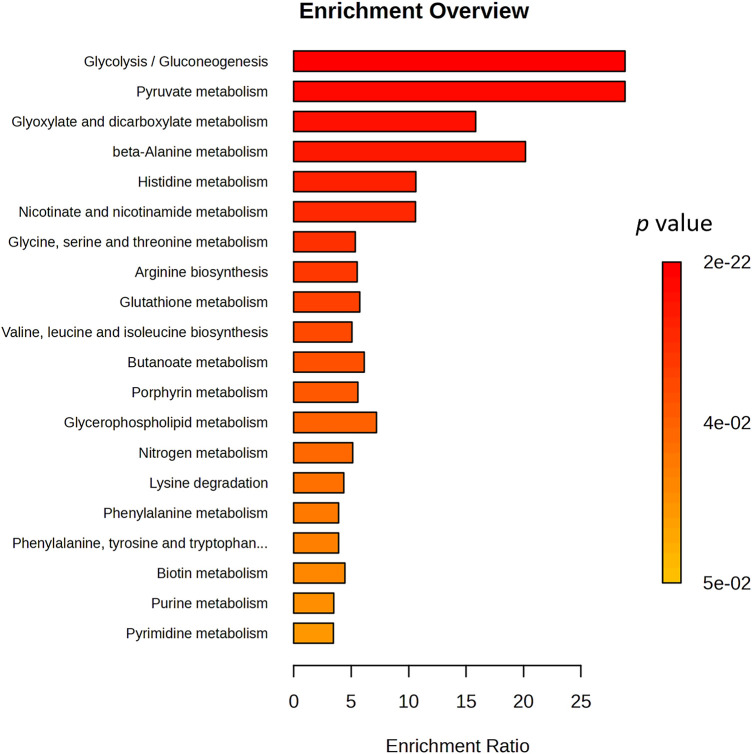
Overview of Enriched Metabolite Sets with significant *p*-value. Bar plot showing significantly enriched metabolic pathways, ranked by enrichment ratio. The color gradient represents the associated *p*-values. Key metabolic pathways such as glycolysis/gluconeogenesis, pyruvate metabolism, and beta-alanine metabolism are highly enriched, suggesting a shift in central carbon metabolism, redox balance, and amino acid turnover

A more general impact of disease on metabolic dysregulation has been evaluated by enrichment pathway analysis of patients at T0 vs. controls (Fig. 5) using Kyoto Encyclopedia of Genes and Genomes (KEGG) pathway [23]. Analysis highlighted how the three most significant pathways, glycolysis, pyruvate and glyoxylate, are driven by acetate and belong to energy metabolism, suggesting metabolic reprogramming in BC following the Warburg effect [13,14,24]. The impact on beta-alanine and histidine pathways, including the downregulation of histidine and aspartate, is also significant. These pathways may be involved in cell stress response, oxidative metabolism, or amino acid turnover, which align with the tumour condition [25,26]. Similarly, the nicotinate pathway is impacted by aspartate downregulation. The Glycine, Serine, and Threonine pathway is conditioned not only by the downregulation of the three aminoacids but also by that of their precursor betaine and choline. Upregulation of glutamate and ornithine is at the base of the impact on arginine and glutathione metabolism. On the other end, pyrimidine and purine metabolism show borderline significance in the analysis; however, their roles are crucial because purine and pyrimidine salvage pathways are well established in cancer, supplying the necessary building blocks for tumor cells to perform DNA and RNA synthesis [13,14].

Following patient profiling at diagnosis, we conducted longitudinal monitoring of metabolites that showed differential expression at baseline in BC patients compared to controls throughout chemotherapy. Our focus was specifically on the subgroup of patients receiving neoadjuvant treatment, as this group provided a larger sample size for a more robust analysis.

In our cohort of patients who received neoadjuvant chemotherapy, 5 achieved partial pathological response (62.5%), 2 complete pathological response (25%), and for 1, the data regarding pathological response is unknown (12.5%). Among these 8 patients, 3 had all three post-baseline samples available for analysis, 3 had two post-baseline samples, and for 2, only the baseline sample was accessible for analysis.

**Figure 6:**
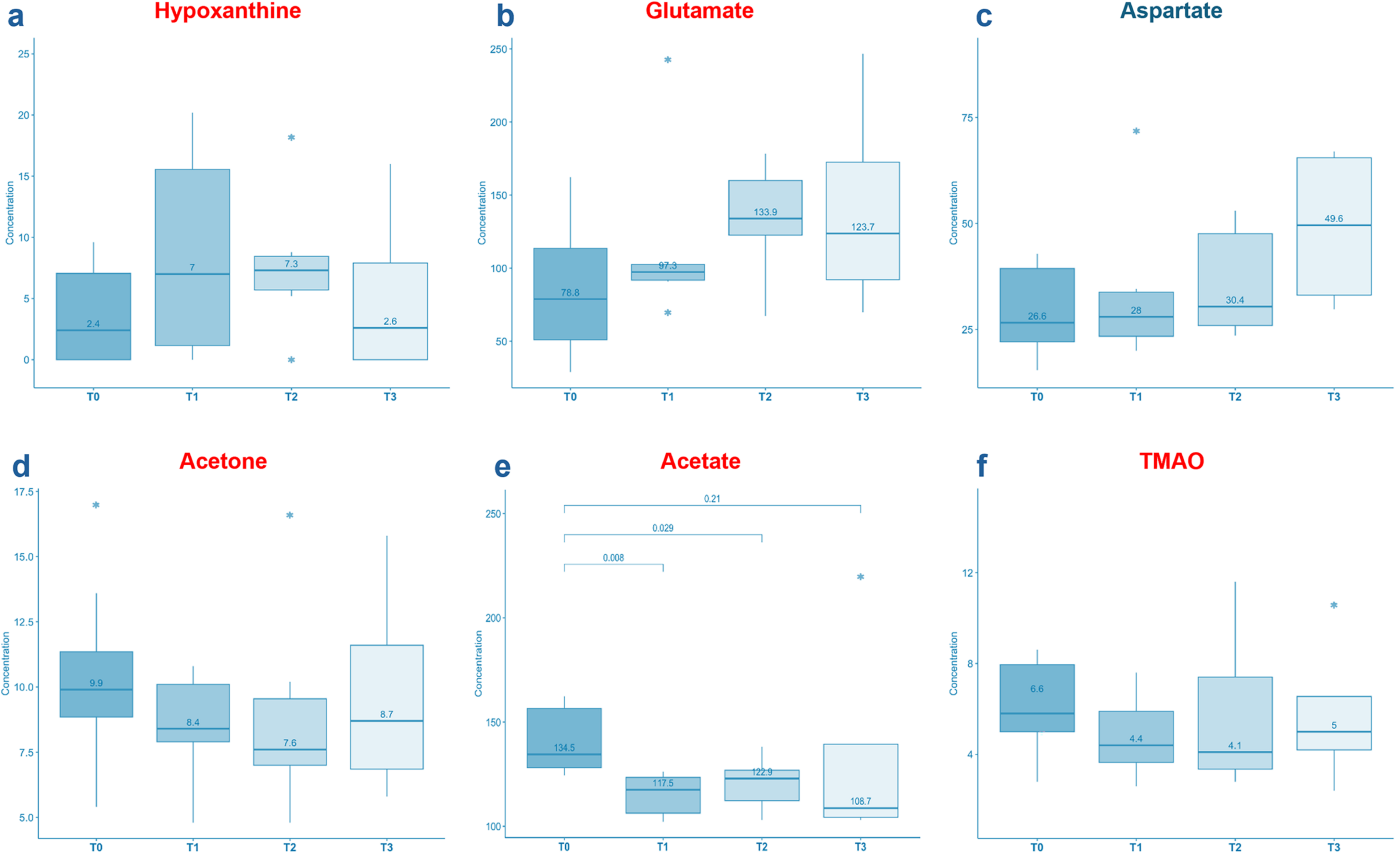
Boxplots showing the variation of some significant metabolites during chemotherapy after 21 days (T1), 42 days (T2) and 63 days (T3) from baseline. The *y*-axis refers to the concentration (µM) of metabolites. Hypoxanthine, glutamate and aspartate displayed a tendency to increase (**a**–**c**). In contrast, acetone, acetate and TMAO exhibited a downward trend (**d**–**f**). Hypoxanthine increasing tendency can be ascribed to cell death and DNA degradation. Acetone, acetate and TMAO decreases suggest a potential predictive role in chemotherapy response. In particular, acetate (reported *p*-values) shows a significant variation during the treatment. The asterisks (*) above and below the boxplots represent the outlier values

When examining the upregulated metabolites at baseline in BC patients, hypoxanthine and glutamate displayed a tendency to increase (Fig. 6a,b), particularly evident in the early cycles of chemotherapy. In contrast, acetone, acetate and TMAO exhibited a downward trend (Fig. 6d–f). Notably, the decrease in acetate was statistically significant (T1 vs. T0 *p* = 0.008; T2 vs. T0 *p* = 0.029; T3 vs. T0 *p* = 0.21), as indicated by the reported *p* values in Fig. 6e. In contrast, aspartate, which showed a downregulated pattern in BC patients at baseline, exhibited an upward trend during treatment (Fig. 6c).

## 4 Discussion

Despite significant progress in BC treatment, the overall prognosis for diagnosed patients remains unfavourable. Therefore, there is an urgent need for easy identification of relevant prognostic and predictive biomarkers to improve the clinical management of BC patients. Metabolomics presents a promising method for discovering potential biomarkers. In addition to diagnostic markers, which allow early detection without invasive procedures, metabolomics also offers opportunities for prognostic and predictive markers. Identifying specific metabolites through metabolomics not only enhances patient phenotyping at the molecular level but also deepens the understanding of the mechanisms behind cancer development.

Overall, the studies conducted thus far have unveiled disruptions in various metabolic pathways crucial for energy production, including glycolysis, the tricarboxylic acid cycle, β-oxidation of fatty acids, carnitine shuttle, and amino acid metabolism [11,12,27]. Elevated glutathione levels are indicative of a response to increased oxidative stress in tumor cells [17], while alterations in glycerophospholipid metabolism reflect changes in membrane biosynthesis processes [18,19,27,28]. Additionally, the over-regulation of purine and pyrimidine metabolism is consistent with the enhanced nucleic acid synthesis commonly observed in cancer cells [15,18,19]. These findings emphasize the complex metabolic reprogramming occurring in the cancer cells and offer valuable insight into potential targets for therapeutic intervention.

In our single-center observational study, serum samples from subjects with BC exhibited higher hypoxanthine levels compared to those of healthy controls. This finding is consistent with previous studies conducted on both *ex vivo* tissue samples and serum samples [15,19]. As is well established, purine synthesis occurs either *de novo* or through a salvage pathway that recycles purine bases, mainly hypoxanthine. The salvage purine synthesis pathway is more ATP-efficient than the *de novo* synthesis, thereby supporting tumor cell growth by consuming less ATP than surrounding normal cells [29]. Indeed, several studies support this observation, demonstrating the upregulation of the enzyme hypoxanthine phosphoribosyl transferase 1 (HPRT1) (that converts hypoxanthine to its nucleotide monophosphate, IMP) in various types of cancer, including BC. Notably, this condition is found to be associated with a worse prognosis [30].

Among the overexpressed metabolites observed in BC patients enrolled in our study were acetate and acetone. The latter is part of the ketone bodies (KB) metabolic group, along with 3-hydroxybutyrate (3HB) and acetoacetate (AcAc), with acetone being a decarboxylation derivative. An increase in the acetone level is an indicator of KB dysregulation. Consistent with our findings, Cao et al. demonstrated that patients with BC had higher serum AcAc levels compared to healthy controls [27]. Ketone bodies, derived from acetyl-coenzyme A via anaerobic glycolysis and fatty acid beta-oxidation, serve as energy sources or precursors for fatty acids [31]. Likewise, elevated acetate levels are associated with increased production of acetyl-Coenzyme A (Acetyl-CoA). Acetyl-CoA is a central metabolic intermediate primarily derived from glucose, glutamine and fatty acids. However, cancer cells’ ability to produce Acetyl-CoA from these conventional carbon sources is dramatically reduced under hypoxia [32,33]. Consequently, cancer cells utilize acetate to generate Acetyl-CoA for lipid synthesis [34]. Besides its role as an immediate metabolic precursor inducing fatty acid synthesis, acetate is also involved in the epigenetic regulation of lipid synthesis and the promotion of cell survival under unfavourable conditions [35]. Elevated lipid metabolism products, including ketone bodies and acetate, may serve as a valuable biomarker for cell proliferation, given the essential role of lipogenesis in cell membrane biosynthesis and subsequent cell growth.

In our study, additional metabolites that were upregulated in BC patients compared to controls include lactate, glutamate, phenylalanine, and ornithine. Several studies using cell lines [12,13], *ex vivo* tissues [36] and serum samples [37] have reported elevated lactate levels in tumor samples compared to controls. Notably, more aggressive cancer cell lines exhibit increased pyruvate consumption, alongside elevated production of lactate and alanine, indicative of an enhanced aerobic glycolytic activity—commonly referred to as the Warburg effect [13,14]. This glycolytic shift, involving the conversion of pyruvate to lactate, is linked to the maintenance of NAD+ levels, which are crucial for tumor glycolytic flux [38]. This data suggests that cancer progression may be associated with alterations in the glycolytic profile, providing valuable insights for potential therapeutic interventions and the identification of biomarkers for disease progression.

Similarly, previous studies involving *ex vivo* tissue, serum, and urine samples have reported elevated levels of phenylalanine [15,36,39,40] and glutamate [36]. These findings may reflect enhanced amino-acid metabolism, a hallmark of cancer progression. Additionally, they suggest that cancer cells may utilize alternative energy sources, such as glutaminolysis, for their growth, proliferation and survival. This process helps replenish the tricarboxylic acid cycle with intermediates diverted for biosynthetic purposes through anaplerotic reactions [41]. These findings underscore the complex metabolic adaptations that occur in cancer cells to meet the demands of rapid growth and proliferation.

We did not observe any statistically significant differences in metabolite concentrations when comparing patients with bladder cancer across different treatment settings—namely, neoadjuvant, adjuvant, and metastatic at baseline. This is likely attributable to the small sample size rather than a true absence of differences among the various disease settings.

To our knowledge, this is the first metabolomics study to assess BC patients receiving chemotherapy in different settings, providing longitudinal monitoring to identify potential alterations in the metabolic profile induced by chemotherapy. In our cohort of patients who received neoadjuvant chemotherapy, 62.5% achieved a partial pathological response and 25% achieved a complete pathological response. In this cohort, an increase in hypoxanthine levels was observed, possibly indicative of cell death and DNA degradation. This hypothesis could be supported by the correlation with uric acid levels. Conversely, acetone, acetate and TMAO concentrations decreased, suggesting a potential predictive role of these metabolites in chemotherapy response, which aligns with the observed patient outcomes. These findings highlight the unique insights that can be gained through longitudinal metabolomic analysis in the context of chemotherapy for bladder cancer. Focusing on the monitoring of these metabolites throughout treatment could further validate their predictive role in chemotherapy response and assist in the earlier identification of non-responding patients, who could then be more promptly directed to surgical resection.

Key limitations of our study include a small sample size, heterogeneity in the enrolled population, and a short follow-up period. In particular, the limited number of patients in specific subgroups—such as those receiving carboplatin-gemcitabine or presenting with metastatic disease—precluded meaningful comparative analyses between treatment regimens and disease stages (e.g., M0 vs. M1). Conducting such comparisons under these conditions would risk generating biased or unreliable conclusions. To ensure methodological rigor, we therefore focused our analyses on the most homogeneous subgroup: patients undergoing neoadjuvant chemotherapy. Future perspectives involve expanding the cohort size and extending the follow-up duration. This strategic approach aims to enhance statistical power and comprehensiveness, allowing for more comprehensive subgroup analyses and enabling the identification of potentially predictive biomarkers for treatment response.

## 5 Conclusions

The results of our pilot study confirm perturbations in several metabolic pathways crucial for energy production in serum samples from patients with bladder cancer, including glycolysis, fatty acid metabolism, amino acid metabolism, and purine metabolism. In addition, TMAO may contribute to the development of urothelial carcinoma by promoting a pro-inflammatory and oxidative stress state. These findings support the potential of metabolic biomarkers for early diagnosis and prognosis assessment in patients with urothelial bladder cancer. Furthermore, monitoring these metabolites may provide a valuable tool for predicting treatment response.

## Supplementary Materials





## Data Availability

The datasets used and/or analysed during the current study are available from the corresponding author on reasonable request.

## Supplementary Materials

The supplementary material is available online at https://www.techscience.com/doi/10.32604/or.2026.068896/s1.

## Abbreviations

3HB: 3-Hydroxybutyrate
AcAc: Acetoacetate
Acetyl-CoA: Acetyl-Coenzyme A
AIOM: Italian Association of Medical Oncology
AJCC: American Joint Committee on Cancer
Ba/Sq: Basal/Squamous
BC: Bladder Cancer
BMI: Body Mass Index
CaG: Carboplatin-Gemcitabine
CG: Cisplatin-Gemcitabine
CNTR: Control
ECOG: Eastern Cooperative Oncology Group
EDTA: Ethylenediaminetetraacetic Acid
FC: Fold Change
HG: High Grade
HPRT1: Hypoxanthine Phosphoribosyl Transferase 1
IMP: Inosine Monophosphate
K2HPO4: Dipotassium Hydrogen Phosphate
KB: Ketone Bodies
KEGG: Kyoto Encyclopedia of Genes and Genomes
KH2PO4: Monopotassium Phosphate
LumNS: Luminal Unspecified
LumP: Papillary Luminal
LumU: Luminal Unstable
MIBC: Muscle invasive bladder cancer
NaOH: Sodium Hydroxide
NMIBC: Non-Muscle-Invasive Bladder Cancer
NMR: Nuclear Magnetic Resonance
oPLS-DA: Orthogonal Partial Least Squares Discriminant
PB: Phosphate Buffer
PROJECT: Periodic Refocusing of J Evolution by Coherence Transfer
PS: Performance Status
Pt-Ct: Platinum-Based Chemotherapy
ROS: Reactive Oxygen Species
sPLS-DA: Sparse Partial Least Squares Discriminant Analysis
TMA: Trimethylamine
TMAO: Trimethylamine N-Oxide
TSP: Trimethylsilylpropionic Acid-d4
