# Supplementary figures and images for: Serum Biomarkers in Bladder Cancer: NMR Metabolomics for Identification and Monitoring during Platinum-Based Therapy
Source: Oncol Res. 2026 Mar 23;34(4):1. doi: 10.32604/or.2026.068896 (PMC13040325; doi:10.32604/or.2026.068896)

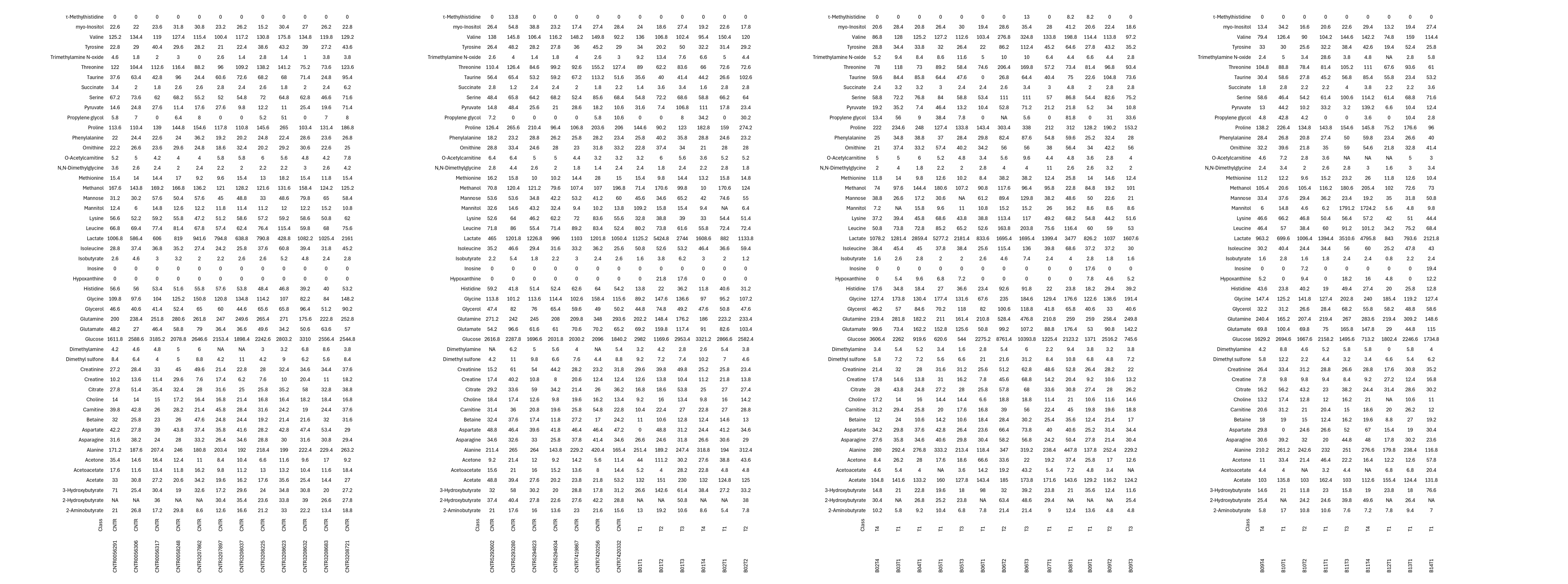

Supplement: Supplementary file 1 [file OncolRes-34-68896-s001.tif]

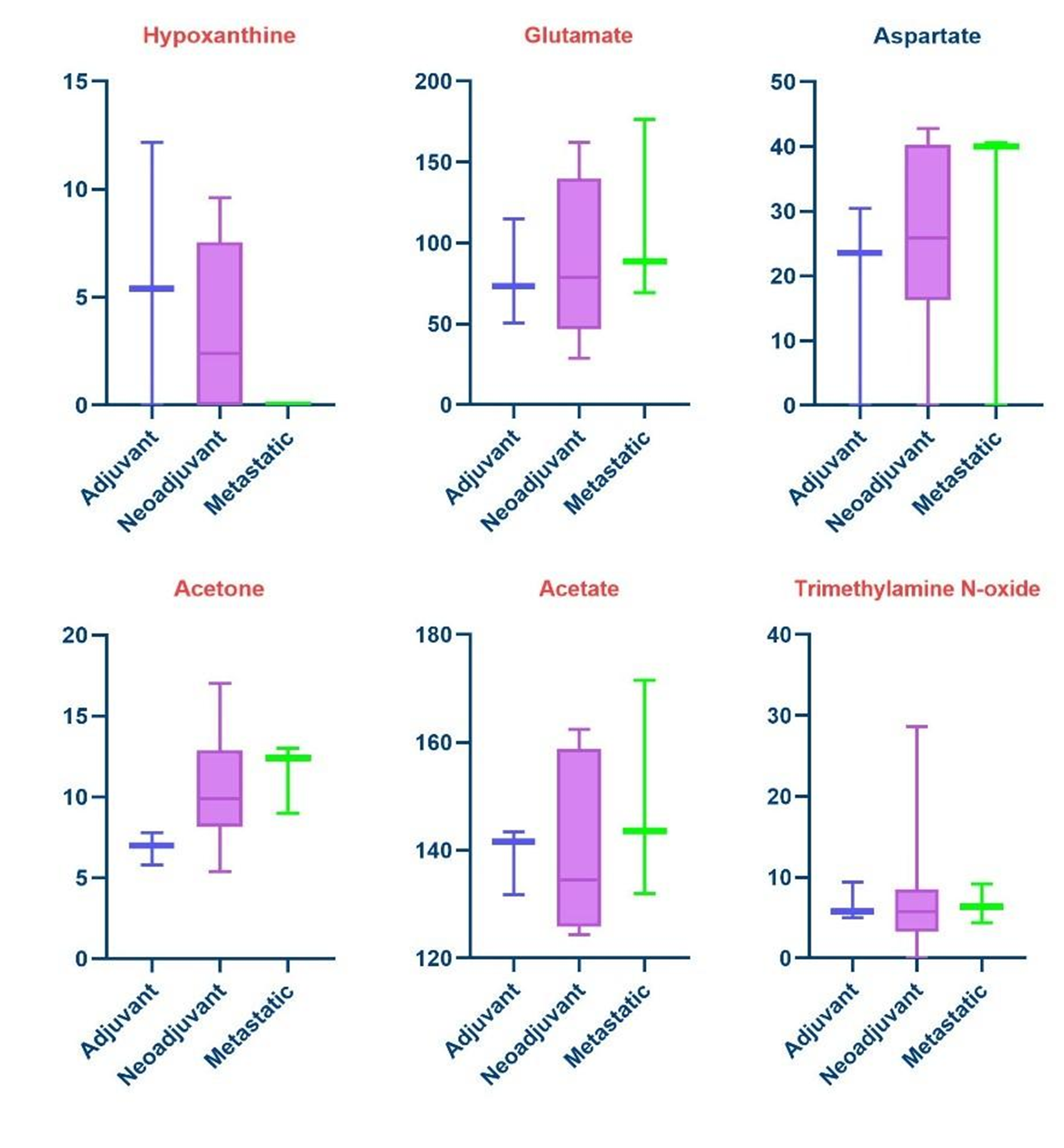

Supplement: Supplementary file 2 [file OncolRes-34-68896-s002.tif]
